# Automatic theranostics for long-term neurorehabilitation after stroke

**DOI:** 10.3389/fnagi.2023.1154795

**Published:** 2023-05-16

**Authors:** Sa Zhou, Jianing Zhang, Fei Chen, Thomson Wai-Lung Wong, Shamay S. M. Ng, Zengyong Li, Yongjin Zhou, Shaomin Zhang, Song Guo, Xiaoling Hu

**Affiliations:** ^1^Department of Biomedical Engineering, The Hong Kong Polytechnic University, Kowloon, Hong Kong SAR, China; ^2^Department of Electrical and Electronic Engineering, Southern University of Science and Technology, Shenzhen, China; ^3^Department of Rehabilitation Sciences, The Hong Kong Polytechnic University, Kowloon, Hong Kong SAR, China; ^4^Beijing Key Laboratory of Rehabilitation Technical Aids for Old-Age Disability, National Research Centre for Rehabilitation Technical Aids Beijing, Beijing, China; ^5^Health Science Center, School of Biomedical Engineering, Shenzhen University, Shenzhen, China; ^6^Key Laboratory of Biomedical Engineering of Education Ministry, Zhejiang Provincial Key Laboratory of Cardio-Cerebral Vascular Detection Technology and Medicinal Effectiveness Appraisal, Department of Biomedical Engineering, School of Biomedical Engineering and Instrument Science, Zhejiang University, Hangzhou, China; ^7^Department of Computing, The Hong Kong Polytechnic University, Kowloon, Hong Kong SAR, China; ^8^Shenzhen Research Institute, The Hong Kong Polytechnic University, Shenzhen, China; ^9^University Research Facility in Behavioural and Systems Neuroscience (UBSN), The Hong Kong Polytechnic University, Kowloon, Hong Kong SAR, China; ^10^Research Institute for Smart Ageing (RISA), The Hong Kong Polytechnic University, Kowloon, Hong Kong SAR, China

**Keywords:** long-term neurorehabilitation, stroke, theranostics, automatic treatment, automatic evaluation, automatic rehabilitation management

## 1. Introduction

Stroke has been a leading cause of permanent motor disability worldwide (World Health Organization, 2013). Long-term neurorehabilitation is required for post-stroke motor functional restoration to increase independence in performing activities of daily living (ADLs) (Mohd Nordin et al., 2014). Given the expanding population of stroke survivors, the long-term neurorehabilitation after stroke has been challenging in the traditional manual-physical therapies, which rely heavily on professional manpower for diagnosis and treatment in a face-to-face manner throughout the rehabilitation process, particularly during a pandemic with tight regulations regarding social distancing, e.g., during the COVID-19 outbreak (Dworzynski et al., 2015; Chen et al., 2022). Self-help telerehabilitation, e.g., at home, through automatic diagnosis and treatment technologies is expected to be an alternative for long-term neurorehabilitation as it provides flexibility to both the therapist and patient by minimizing the close physical contact when necessary and the professional manpower demand in a post-pandemic future (Hosseiniravandi et al., 2020; Nam et al., 2021). Effective theranostics after stroke requires quantitative measurements for efficient evaluation, intensive and regular treatment delivery, and rehabilitative management in long term. However, theranostic automation for stroke rehabilitation, i.e., diagnosis, treatment, follow-up, and prognosis, has not been well-developed yet. This article provided opinions on recent advances and future directions in the automation of long-term neurorehabilitation services after stroke. We examined the existing gaps in available technologies that have the potential to support automatic theranostics at the upper layer of application. First, we discussed quantitative measurements using neuroimaging and kinematic/kinetic technologies for post-stroke neurobehavioral evaluations. Second, we discussed automation of rehabilitation treatments with coordination between the patient and therapist in terms of advances in home-based telerehabilitation and its related robotic technologies to support the self-help physical training under unconventional environments. The fundamental requirements of the robotic technologies in digitalized physical and cyber networks for telerehabilitation are also noted. Finally, we discussed the integration of social interactions into the physical and cyber worlds for smart poststroke rehabilitation management in the Industry 4.0 era.

## 2. Automation of neuro-behavioral measurements and their correlations with clinical assessments

Longitudinal evaluation is important for monitoring rehabilitative progress and adjusting the interventions for optimal clinical outcomes in neurorehabilitation after stroke (Porciuncula et al., 2018; Mehrholz, 2019). Multimodal measurements of the hemodynamics, neurodynamics, and kinematics/kinetics in neuro-behavioral impairments have been advocated for effective post-stroke evaluation; this is because behavioral outcomes with substantial heterogeneities across patients were found to be a result of the synergistic effects from the central (e.g., inter-hemispheric asymmetry and corticospinal tract integrity) to peripheral nerves systems (e.g., muscle synergy and range of motion) (O'Dwyer et al., 1996; Wegrzyk et al., 2017; Thrane et al., 2020). Traditional clinical assessments with total reliance on individual experience-based diagnoses, e.g., Fugl–Meyer Assessment (FMA) for sensorimotor evaluation, were widely accepted by clinical practitioners because of the operational simplicity of manual evaluation as well as the holistic and intuitive scales on multifunctional impairments, including sensorimotor, cognitive, and emotional deficits after stroke (Cheung et al., 2018; Zhou et al., 2021a). However, clinical assessments were rarely performed frequently or even irregularly in long-term rehabilitation programs, which was constrained by short-handed rehabilitation services (Pumpa et al., 2015). The evaluation accuracy may be affected by the low inter-assessor repeatability when changing assessors inevitably in long-term programs because of the subjectivity and insensitivity to subtle behavioral changes and the indirect neurological assessments under clinical conditions (Pumpa et al., 2015; Zhou et al., 2021b). In contrast to the traditional assessments, quantitative measurements of the neuro-behavioral changes via neuroimaging and kinematics/kinetics technologies, e.g., magnetic resonance imaging (MRI) and motion capture systems, provided objective and sensitive solutions for post-stroke functional evaluation (Hu et al., 2022). However, one of the challenges regarding the application of quantitative measurements to automatic assessments is the operational complexity of the measurement system that relies heavily on professionals, particularly for multimodal measurements that employ various standalone systems (Nazarova et al., 2020). The operational complexity also poses considerable challenges to self-help rehabilitation, which requires easily operable and compact devices for operation by nonprofessionals such as caregivers and the patients themselves, with quantitative measurement systems in unconventional home-based or even outdoor settings (Nam et al., 2020). For example, neurological measurements on cerebrovascular activities with high spatial resolution in clinical practice, such as computed tomography (CT) and MRI, required professional operation of large and expensive equipment, resulting in limited availability for long-term service (Shahrestani et al., 2021). Similar complex operations were required for visual marker-based optoelectronic motion capture systems as the gold standard in kinematics/kinetics measurements, which was impractical even in a clinical setting owing to the large setup volume and high cost (Mesquita et al., 2019). Among the next-generation technologies that can be potentially used in point-of-care neurological diagnoses, including functional near-infrared spectroscopy (fNIRS), electroencephalography (EEG), and portable MRI devices, EEG has been reported as the most promising approach for automatic long-term neurorehabilitation assessments because of its high temporal resolution, low cost, and safe operation (Shahrestani et al., 2021; Guo et al., 2022). Nonetheless, multichannel EEG systems used to evaluate post-stroke cortical reorganization typically required professionals for setting up the massive systems to ensure adequate signal quality, including mounting the headset onto the corresponding cortical areas and preparing the electrodes for appropriate electrode–skin impedance (e.g., <5 kΩ) (Shahrestani et al., 2021; Zhou et al., 2021a,b). Higher operational complexities with extensive setup were common in multimodal measurements for simultaneous data acquisition from different systems, e.g., >30 min of system setup for capturing corticomuscular coupling based on EEG and electromyography (EMG) (Guo et al., 2022). Bimodal integration of measurement systems has also emerged for simplifying the standalone system setup and leveraging the complementary strengths of different measurement modalities, e.g., EEG-fNIRS system for high spatial–temporal resolution neuroimaging (Sangtae and Jun, 2017). Encouraging preliminary results have been achieved for functional independence assessment after stroke using the bimodal EMG and inertial measurement unit (IMU) system with highly integrated and wireless sensors for monitoring the ADLs in a well-controlled lab environment (Mouawad et al., 2011). Despite the progress achieved in bimodal integration, the target users of most measurement systems are still limited to professionals in either clinical or lab environments rather than nonprofessionals in self-help rehabilitation (Shahrestani et al., 2021). Hence, further research, such as translational study and commercialization, is needed on hybrid neuro-behavioral evaluation systems with highly integrated and easily operable multimodal measurements from both the central and peripheral nervous systems for automatic poststroke assessments in the future.

In addition to simplifying the operational complexities of the measurement facilities, straightforward diagnostic metrics are required from quantitative measurements for early identification of deviations from the desired progressive changes in long-term rehabilitation (Sarmento et al., 2019). Compared to the clinical scales, neurological and kinematic/kinetic metrics from quantitative measurements provided detailed information on the neurobehavioral changes with novel biomarkers as the therapeutic targets for potential precise rehabilitation (Cheung et al., 2012). However, challenges remain with respect to the low acceptance of most metrics containing technical details by clinical practitioners, such as neuroimages and mathematical parameters, which typically rely on experienced specialists with engineering backgrounds for interpretation (Sarmento et al., 2019). For example, the manual interpretation of neuroimages for feature extraction of the post-stroke impairments required cross-disciplinary collaborations among specialists for accurate evaluation, leading to substantial time delays in intervention planning by the therapists (Sarmento et al., 2019). There are inconsistencies in the bio-signal processing pipelines among specialists for neural decoding, particularly for visual inspection and manual rejection of artifacts in the preprocessing of raw signals, such as the blood oxygen level dependent (BOLD)-fMRI and EEG recordings (Rajkumar et al., 2018; Sarmento et al., 2019). In addition, the most sensitive metrics that have high responsiveness to rehabilitative progress for monitoring and predicting rehabilitation outcomes in long-term programs remain uncertain despite the validities of various metrics from quantitative measurements were demonstrated by their correlations with clinical scores (Cheung et al., 2012). This has also hindered the translation of the metrics from quantitative measurements to precise therapeutic targets for automatic intervention planning (Cheung et al., 2012). A variety of feature integration and dimension reduction algorithms has been employed to improve the readability of various metrics from quantitative measurements (Xie et al., 2018; Cheung and Seki, 2021), such as the neurovascular and corticomuscular coupling metrics for bimodal integration of the respective fNIRS-EEG and EEG-EMG signals (Lou et al., 2013; Zhou et al., 2018; Shahrestani et al., 2021). Promising results have been achieved with the corticomuscular coupling metric for monitoring the post-stroke evolutions of integrated central-and-peripheral voluntary motor efforts in the target muscles in a 20-session rehabilitation program, which demonstrated its potential responsiveness in longitudinal evaluations (Guo et al., 2022). Despite the progress made to date, large amounts of data from multimodal measurements have posed challenges to clinical practitioners in long-term rehabilitation programs (Ye et al., 2021). Further investigations of the straightforward diagnostic metrics with high responsiveness to rehabilitative progress are thus needed for multimodal measurements in future longitudinal studies.

As large amounts of data are generated during measurements for automated evaluation during self-help poststroke rehabilitation, machine learning (ML) methods have emerged as promising assistive techniques to healthcare providers who wish to rapidly analyze batches of data as well as project the neuro-behavioral metrics onto clinical assessment scores for readable results (Abraham et al., 2014; Kabade et al., 2021). Using the neuro-behavioral metrics and neuroimages as input features, the pretrained ML-based models can automatically distinguish differences between stroke patients and unimpaired participants. It has been proven that compared to the readings of human professionals, ML-based models could significantly reduce the evaluation time required to determine symptom onset related to stroke (Chae et al., 2020). In addition, the output predictions of the trained models with ML algorithms, such as random forest, support vector machine (SVM), and convolutional neural network (CNN), have been observed to correlate with clinical scores (Moghadam et al., 2022; Sung et al., 2022; Zhang et al., 2022). There were significant correlations between the outputs of the ML-based models and the manual results by human professionals, which has increased the readability of performance of the ML-based models (Ye et al., 2021). Although ML has advantages in speeding up the process with batches of data, one of the main obstacles is the robustness of the ML-based model (Cui et al., 2020). Robust predictions by ML models require high homogeneity of the input data. However, individual stroke patients often have multimodal input data, e.g., text, images, and voice, collected over long-term evaluations, and such data heterogeneity may affect the model robustness (Lum et al., 2002). Meanwhile, it is difficult for individuals who have suffered a stroke to independently operate complex equipment for collecting and processing raw data before applying to ML-based models. These obstacles could be addressed by designing easy-to-operate diagnosis equipment with one-touch operation or automatic device calibration before they are operated by stroke patients. Additionally, several algorithms and software were integrated into the equipment to automatically coordinate and analyze raw multimodal data, thereby promoting robust prediction by the ML-based model (Park et al., 2019; Pedroni et al., 2019; Rosero-Rodríguez and Alfonso-Morales, 2021).

## 3. Automation in rehabilitation treatments with coordination between patient and therapist

Effective motor restoration after stroke requires intensive physical training of the paralyzed limb with maximized voluntary motor effort (VME) and minimized compensatory motions, in addition to the updated intervention plans with precise therapeutic targets from neurobehavioral monitoring in long-term neurorehabilitation (Hu et al., 2006; Guo et al., 2022). Conventional center-based rehabilitation treatments constrained the availability and accessibility of rehabilitation services to discharged patients who required regular and intensive physical therapy due to patients' transportation difficulties with the reduced mobility and the rehabilitation centers' resource constraints even in developed countries (Sarfo et al., 2018). Home-based telerehabilitation with remote supervision by professionals has thus emerged as an alternative mode of regular physical treatment after discharge in the long term, particularly post the COVID-19 outbreak and its restrictions on social distancing (Hosseiniravandi et al., 2020; Nam et al., 2021). However, there are also challenges with respect to limited or uncertain rehabilitation effectiveness in most self-help rehabilitation technologies, such as the Kinect and tablets with rehabilitative games, virtual reality (VR), and self-help rehabilitation robots, for assistance with home-based telerehabilitation (Chen et al., 2013; Hosseiniravandi et al., 2020). Despite the convenience and high accessibility of the Kinect, tablets, and VR technologies for decentralized rehabilitation training, they were lacking the necessary physical assistance for patients to relearn the desired movements, resulting in limited rehabilitation effectiveness (Nam et al., 2021). Several self-help rehabilitation robots, e.g., HandSOME (Chen et al., 2017), MyoPro (Mccabe et al., 2019), and the EMG-driven exoneuromusculoskeleton (Nam et al., 2021), have emerged in recent works to provide physical assistance and alleviate the labor-intensive process in repetitive limb practice after stroke; these systems offered advantages of high intensity and low cost for long-term home-based telerehabilitation (Nam et al., 2021). Promising rehabilitation effectiveness was achieved for upper limb voluntary motor function with the EMG-driven exoneuromusculoskeleton in pilot clinical trials, which could be attributed to the recruitment of VME in the target muscles based on the EMG signals compared to the continuous passive movement mode in most self-help rehabilitation robots (Nam et al., 2021). Despite the progress achieved with self-help rehabilitation robots, challenges remain regarding the lack of rehabilitative monitoring on neurobehavioral changes in home-based telerehabilitation (Shahrestani et al., 2021). Little was known about the anticipated dosage and rehabilitative plateau in treatment planning with the self-help rehabilitation robots (Abduallah et al., 2007), where compensation from the unaffected hemisphere and proximal muscles (e.g., elbow, shoulder, and body trunk) could be possibly induced without precise therapeutic targets from the rehabilitative monitoring (Zhou et al., 2021a). In future home-based telerehabilitation, multimodal measurements on the neurobehavioral changes should be incorporated into the self-help rehabilitation robots for optimal clinical outcomes.

In addition to the self-help rehabilitation devices, effective remote management is important for coordination between the patient and therapist so as to guarantee constant and regular physical training with sufficient intensity in home-based telerehabilitation (Hosseiniravandi et al., 2020). Despite the promising outcomes of home-based telerehabilitation from well-controlled clinical trial studies (Gregory et al., 2011), their feasibility of translation into clinical routine remains a question given the difficulties of remotely implementing technical guidance and support for using self-help rehabilitation devices as well as supervising the training progress, which could bias the rehabilitation effectiveness achieved in the original clinical trials (Hosseiniravandi et al., 2020; Podury et al., 2021). For example, professional guidance was still needed in the clinic to help patients grasp device operation before self-help training with the rehabilitation robots; for instance, a total of three and 12 supervised tutorial sessions (60 to 90 min per session) were delivered to the stroke patients in previous studies with the EMG-driven exoneuromusculoskeleton and MyoPro, respectively (Gregory et al., 2011; Nam et al., 2021). Meanwhile, time-consuming technical support to restore malfunctioning devices in home-based rehabilitation could reduce patient engagement, leading to a high drop-out rate in long-term training programs (Sarfo et al., 2018). Furthermore, a lack of adherence to the prescribed rehabilitative protocol with varied training durations and frequencies among individuals could occur because of the lenient supervision of training progress, such as the training duration ranging from 2 to 60 min per day (Nijenhuis et al., 2016). In this regard, the Internet of Things (IoT) technology has been employed for remote management of the rehabilitative progress by bridging the gap between the patients and therapists via interconnected telerehabilitation devices with embedded sensors, which could potentially promote patients' engagement in home-based physical training (Hosseiniravandi et al., 2020). Promising rehabilitation effectiveness of the IoT-assisted home-based telerehabilitation has been reported in recent randomized controlled clinical trials, where augmented improvements in the upper limb motor functions, e.g., range of motion (ROM) of the wrist joint, were found with the IoT-assisted tenodesis-induced-grip exoskeleton robot compared to conventional task-specific motor training (Hsu et al., 2021). Despite the promising outcomes achieved so far, challenges still remain for the low efficiency of coordination between patients and professionals in IoT-assisted telerehabilitation, where substantial time delays for intervention planning could occur in data management and analytics that rely on professionals (Sarmento et al., 2019). In this respect, AIoT has emerged for more efficient IoT operations via enhanced data management and analytics with artificial intelligence (AI) algorithms (Lai et al., 2021). Despite the wide application of AIoT in smart cities, smart retail, and smart appliances, little has been done for implementing AIoT in telerehabilitation management, possibly owing to the immaturity of current theranostic systems for self-help rehabilitation (Lai et al., 2021). For future automation in rehabilitation treatment, AIoT could be incorporated with novel point-of-care diagnostics and treatment devices to augment the efficiency of telerehabilitation management for real-time decision making in intervention planning.

## 4. Automation in coordination of healthcare resources in long-term rehabilitation services

Besides the automation in stroke diagnosis and treatment discussed earlier, long-term rehabilitation services after stroke also require automatic management of the healthcare resources, including human resources, medical devices, and information from the theranostic process (Dworzynski et al., 2015; Zelenák et al., 2021). An automation platform for coordinating the healthcare resources could thus help alleviate the workload of human professionals, optimize the use of medical devices, and facilitate theranostic effectiveness (Jadczyk et al., 2021; Poonsuph, 2022). Conventional management of poststroke rehabilitation services relies heavily on manpower and manual interactions, where cross-disciplinary professionals (therapists, nursing staff, psychologists, etc.) interact with the stroke patients regularly via hands-on or face-to-face interventions to support the patients in the rehabilitative program through a center-based service (Dusenbury and Alexandrov, 2020; Jain and Chatterjee, 2020; Saverino et al., 2021). However, given the anticipated increase in medical needs for stroke rehabilitation, current poststroke human resources and medical devices are limited by challenges such as shortage and uneven physical distribution in local institutions (Yuehong et al., 2016). Meanwhile, vast amounts of information obtained from long-term rehabilitation services are always dynamic and heterogeneous, relevant to the diversity of individual patient status and progress, which makes the current management system a bottleneck for achieving timely, individualized, and optimized treatment (Frontera et al., 2017).

**Figure 1:**
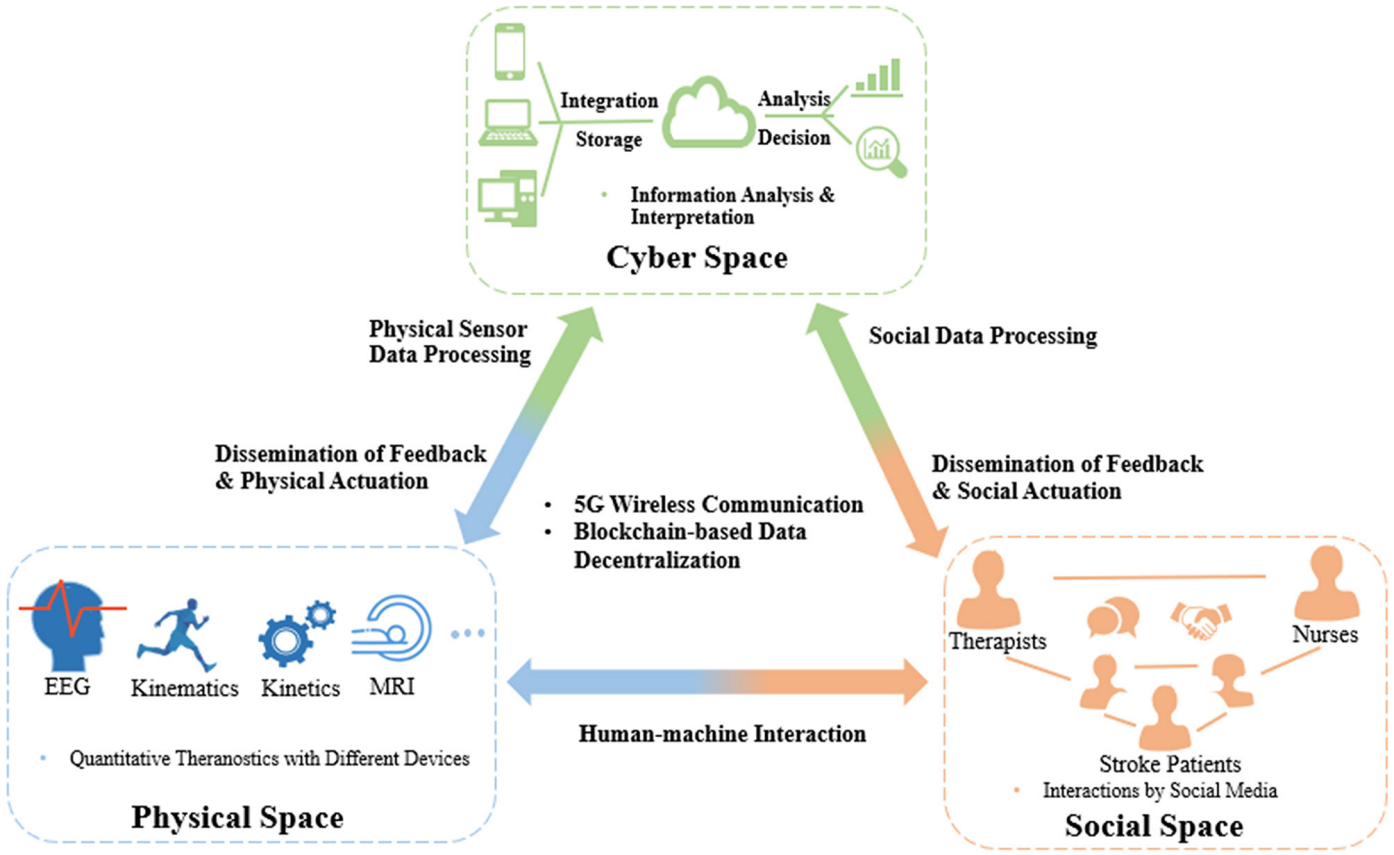
CPSS platform for coordinating healthcare resources in long-term rehabilitation services after stroke.

With the development of modern mobile communication and data technologies, IoT-based cyber-physical system (CPS) has been proposed in various domains to realize real-time, safe, and dynamic collaborations with physical systems (Liu et al., 2017). CPS could seamlessly connect devices between the physical and virtual worlds by identifying, sensing, computing, and actuating physical systems (Reine et al., 2021; Yilma et al., 2021). This architecture has been used for various scenarios, such as energy resource distribution, smart manufacturing, and intelligent transportation (Xiong et al., 2015; Yin et al., 2020; Wang et al., 2022). There was also a report of an attempt to apply this architecture to manage healthcare resources in post-stroke rehabilitation, e.g., a wearable measurement system for monitoring cardiovascular activity in a stroke patient's daily life setting, called as Big-ECG, which was capable of tracking cardiac signals, analyzing data on a cloud platform, and providing health suggestions and messages to assist the patients (Hussain and Park, 2021). However, most of these applications were still immature for poststroke rehabilitation management in individual stroke patients, e.g., no integrated IoT sensors for multimodal evaluation as mentioned earlier, scarcity of social interactions either among the stroke peers for competition and collaboration or between the patients and therapists for professional guidance during physical training (Ventura et al., 2019). Findings on CPS adaptation have shown an emerging trend of adding an additional social layer to the CPS architecture to address both human and social factors, which showed the growing importance of involving social interactions in the CPS (Musil et al., 2017; Sabou et al., 2018). In this regard, a cyber-physical social system (CPSS) was introduced to provide a new paradigm for automatic operation of real-world systems in which the cyber, physical, and social factors are comprehensively considered for decision making in application scenarios (Wang et al., 2022). However, in current healthcare management systems on long-term poststroke rehabilitation, very few studies focus on the influence of social interactions. A possible CPSS platform for automation maximization in coordinating healthcare resources is illustrated as an example in Figure 1.

CPSS platforms will be composed of interactions among the three spaces, i.e., cyber, physical, and social spaces, of which the first is cyber-physical space coupling. The large amounts of theranostic data on stroke patients, e.g., EEG, kinematic, and kinetic parameters, can be dynamically transferred to a distributed medical data cloud integrating various technologies, such as blockchain (Xie et al., 2021), to improve the transparency, immutability, and anonymity of data. Based on the encrypted data, edge-AI-based big data analytics (Himeur et al., 2022) can be performed in the cyberspace to extract interpreted patterns to timely monitor and control the statuses of medical devices and instruments, i.e., dissemination of feedback and physical actuation. The second coupling is that of the social-physical space; this will concentrate on social communications between humans and machines, e.g., incorporating socially assistive robots into the poststroke rehabilitation process, which offers interactive tasks (competitive and/or cooperative) to increase the motivation and training intensities of stroke patients in the long term. The third coupling is of the cyber-social space. The human and social information representing thinking, cognition, and knowledge from social relations, such as therapist–patient, nurse–patient, and therapist–nurse, will be integrated into and exchanged in the cyberspace, similar to the cyber-physical coupling process. After automating big data analytics, the cyberspace will disseminate feedback and social actuation to the social components, e.g., therapists, nurses, and individual stroke patients, via social sensors (e.g., smartphones, smart mobile devices).

## 5. Conclusions and future perspectives

This article provided opinions on the future directions of automatic theranostics for long-term neurorehabilitation after stroke, which mainly depends on the technological breakthroughs in the following aspects, as summarized in Table 1. (1) Evaluation: a hybrid neuro-behavioral evaluation system with multimodal measurements, straightforward diagnostic metrics, and robust prediction of ML-based models is required for future self-help evaluations in unconventional environments. (2) Treatment: an AIoT-assisted home-based telerehabilitation system integrating novel point-of-care diagnostics and self-help robotic devices is required to augment intervention planning, rehabilitation effectiveness, and management efficiency. (3) Healthcare resource management: a modern digitized CPSS platform integrating IoT sensors for multimodal evaluations and social interactions is required to maximize automation in the future coordination of healthcare resources. In conclusion, it is time to implement poststroke rehabilitation theories and expertise from clinical practice into automation infrastructures based on multidisciplinary advances for better long-term neurorehabilitation in the Industry 4.0 era.

**Table 1:** Existing techniques, limitations and knowledge gaps, and future directions on automatic theranostics for long-term neurorehabilitation after stroke.

**Automation of long-term neurorehabilitation services after stroke**		**Current techniques**	**Limitations and knowledge gaps**	**Future direction**
Neuro-behavioral evaluation	Quantitative measurements	Neuroimaging and kinematics/kinetics technologies (Shahrestani et al., 2021): •CT •MRI •Motion capture systems •EEG •fNIRS	Complex operations Inapplicable for self-help evaluation (Shahrestani et al., 2021)	Hybrid neuro-behavioral evaluation systems •High integration •Easy operation •Multimodal measurements
	Diagnostic metrics	Neurological and kinematic/kinetic metrics, e.g., neuroimages and mathematical parameters (Cheung et al., 2012)	Low acceptance by clinical practitioners with (Sarmento et al., 2019) •Manual interpretation by engineering specialists •Inconsistency in the bio-signal processing pipelines •Uncertain sensitivity to rehabilitative progress •Large dataset from multimodal measurements	Straightforward diagnostic metrics with high responsiveness to rehabilitative progress
	Correlations with clinical assessments	ML algorithm prediction with pre-trained model (Abraham et al., 2014)	Low model robustness due to high inhomogeneity of the input multimodal data (Lum et al., 2002)	Easy-to-operate diagnosis equipment with one-touch operation Integrating algorithms and software into the equipment to coordinate raw multimodal data
Rehabilitation treatment	Self-help rehabilitation technologies	Self-help rehabilitation robots, Kinect and tablets with rehabilitative games, and VR …. (Hosseiniravandi et al., 2020)	Limited or uncertain rehabilitation effectiveness (Hosseiniravandi et al., 2020) Unknown anticipated dosage and rehabilitative plateau (Shahrestani et al., 2021)	Incorporating multimodal neuro-behavioral measurements into self-help rehabilitation technologies
	Coordination between patients and therapists	Home-based telerehabilitation (Gregory et al., 2011)	Difficulties of remote management in training supervision, guidance, and technical support Inefficient coordination between patients and professionals (Hosseiniravandi et al., 2020; Podury et al., 2021)	Incorporating AIoT with novel point-of-care diagnostics and treatment devices for telerehabilitation management
Healthcare resources management	Coordinating the human resources, medical devices, and information	Conventional management with manpower and manual interactions (Zelenák et al., 2021) IoT-based CPS platform (Liu et al., 2017)	Resources shortage and uneven physical distribution (Hosseiniravandi et al., 2020) Dynamic and heterogeneous information (Frontera et al., 2017) No integrated IoT sensors for multimodal evaluation (Ventura et al., 2019) Scarcity of social interactions (Liu et al., 2017)	Highlighting social interaction with novel CPSS platform, e.g., peer competition training, coordination of interdisciplinary team approach, guidance of healthcare givers to embrace the new technologies

VR, virtual reality; CT, computed tomography; MRI, magnetic resonance imaging; EEG, electroencephalography; fNIRS, functional near-infrared spectroscopy; ML, machine learning; AIoT, artificial intelligence of things; CPS, cyber-physical system; CPSS, cyber-physical social system.

In addition to the technical automation in neuro-behavioral measurements, rehabilitation treatments and coordination of healthcare resources mentioned above, further advances in the following social aspects are parallelly important to facilitate the automatic theranostics for long-term neurorehabilitation after stroke:

1) Cross-disciplinary education to clinical practitioners on using the automatic theranostic technologies will improve efficiency by enabling a seamless connection between patients and therapists in the long-term service. Current theranostic processes rely heavily on cross-disciplinary collaboration among experienced specialists with distinct clinical and engineering backgrounds, leading to substantial time delays and high costs in not only the provision of healthcare resources but also the clinical translation of cutting-edge theranostic technologies, e.g., the low acceptance of neurobehavioral metrics and self-help rehabilitation technologies by clinical practitioners. When the technical barriers among different disciplines can finally be crossed by automations in the future, the service providers at the frontier, i.e., the practitioners, need to prepare well for the new paradigm.
2) Facilitation of telerehabilitation to rural and under-developed areas with needed quality is an advantage of the automatic theranostics after stroke for achieving equity in receiving long-term services by different populations. The infrastructures of telerehabilitation can provide cost-effective and timely connections between the professional and patient in diagnosis, treatment, and follow up without the constraint of their physical locations, interfaced mainly with the point-of-care IoT systems, e.g., a self-help rehabilitation robot. There will be no significant differences in the quality of the tele-service provided by the same professional and received by patients in different cities.

## Author contributions

SZho and JZ contributed to the conception of the work and manuscript drafting. FC, TW, SN, ZL, YZ, SZha, and SG contributed to the conception of the work. XH conceived of the study and coordinated the whole project, including the conception of the work, and manuscript revising. All authors read and approved the final manuscript.
